# The feasibility analysis of omission of elective irradiation to level IB lymph nodes in low-risk nasopharyngeal carcinoma based on the 2013 updated consensus guideline for neck nodal levels

**DOI:** 10.1186/s13014-017-0869-x

**Published:** 2017-08-18

**Authors:** Xiaomin Ou, Yibing Miao, Xiaoshen Wang, Jianhui Ding, Xiayun He, Chaosu Hu

**Affiliations:** 1 Department of Radiation Oncology, Fudan University Shanghai Cancer Center, Shanghai, China; 2 Department of Oncology, Shanghai Medical College, Shanghai, China; 3 Department of Diagnostic Radiation, Fudan University Shanghai Cancer Center, Shanghai, China

**Keywords:** Nasopharyngeal carcinoma, Elective nodal irradiation, Submandibular gland, Xerostomia, Late toxicity

## Abstract

**Background:**

Level IB metastasis is rare in nasopharyngeal carcinoma (NPC). The purpose of this study is to investigate the high-risk factors for level IB metastasis and evaluate the feasibility of omission of elective irradiation to level IB in the low-risk subgroups in NPC.

**Methods:**

This retrospective study identified 532 patients with NPC treated by definitive radiation in our institution from 2009 to 2010. Level IB nodes were electively irradiated based on the physician’s decision. Diagnostic head and neck MRIs were reviewed. The involvements of nodal levels were evaluated according to 2013 updated guidelines of RTOG. The correlations of level IB metastasis and other factors were studied using Chi-square test and logistic regression model. Log-rank tests were used to compare survival rates. Cox proportional-hazards models were used to evaluate the effect of various factors. Patient-reported xerostomia was recoded in every follow-up and the extents of delayed xerostomia at 1 year post-radiation were compared between those with/without elective level IB irradiation.

**Results:**

N stage, bilateral nodal metastasis, level II involvement, level IIA involvement, level IIA with multiple levels involvement, maximal axial diameter (MAD) of level IIA nodes > 20 mm, MAD of neck lymph nodes > 30 mm, necrosis of level IIA nodes, extracapsular spread of level IIA correlated with level IB metastasis by univariate analysis. In multivariate analysis (MVA), bilateral nodal involvement, MAD of level IIA nodes > 20 mm or extracapsular spread of level IIA nodes, were independent predictive factors for level IB metastasis. Patients without either these factors were denoted low-risk group and the rest high-risk group. Of the low-risk group, there was no significant difference of regional control and overall survival (OS) between those with or without elective irradiation. The percentage of level IB recurrence of those without elective irradiation was 0.46%. Elective level IB irradiation was not significant upon MVA both for regional control and OS. Of the high-risk group, elective level IB irradiation was marginal significant for regional control, but not for OS upon MVA. No regional recurrence located at level IB. Overall, omission of elective irradiation to level IB reduced the mean doses of submandibular glands, but did not improve patient-reported xerostomia.

**Conclusion:**

For patients without high-risk factors of level IB metastasis, omission of elective level IB irradiation did not impair regional control and OS in NPC.

**Electronic supplementary material:**

The online version of this article (doi:10.1186/s13014-017-0869-x) contains supplementary material, which is available to authorized users.

## Introduction

The 5-year overall survival of nasopharyngeal carcinoma (NPC) has achieved 84% of non-metastatic cases with the use of intensity-modulated radiation therapy (IMRT) and optimal chemotherapy [1–4]. Since the survival is prolonged, the late toxicities merit more attention. The most frequently observed late complication was xerostomia. 39.3–40.1% [4–6] of patients reported xerostomia at more than 1 year post-radiation. Sparing of saliva glands is crucial to reduce xerostomia. IMRT is superior to conventional two-dimensional radiotherapy in preserving parotid function and results in less severe delayed xerostomia [6]. Parotid-sparing IMRT for NPC preserved half of parotid excretion compared with baseline [7]. However, sparing of parotid alone has increased salivary flow rate [8], but inconsistently translated to improvement of patient-reported xerostomia [6, 8–10].

Parotids produce most saliva under stimulation, while the submandibular glands (SMG) contribute to the majority of unstimulated saliva [11]. SMG, together with minor salivary glands in the oral cavity, produce mucins that maintain subjective perception of hydration [10]. It is reported that SMG salivary flow rate depends on mean dose and recovers over time up to a threshold of 39Gy [12]. Restriction of mean dose of SMG less than 39Gy helps to improve observer–reported and patient-reported xerostomia [9, 12, 13].

However, the SMG is often included in the target volume of NPC when level IB receives radiation. According to the protocol of RTOG 0225, clinical target volume (CTV) encompassed unilateral level I to level V. The routine of Prince of Wales Hospital in Hong Kong is to electively irradiate level IB to V with a total dose of 54-60 Gy for node-negative cases [14]. However, a number of institutions in mainland of China [4, 11, 15] do not recommend routinely including level IB in the irradiation volume. Currently, there is no consensus of criteria on elective irradiation of level IB. Zhang et al. [11] has recently demonstrated that level IB-sparing IMRT should be feasible for patients without high risk factors for level IB metastasis. However, in this study, only a few factors were included in the analysis, such as T, N, level IIA and retropharyngeal metastasis, et al. More clinical characteristics should be incorporated in to the analysis for level IB metastasis, especially the details of involvement of various neck nodal levels. Hence, we performed this retrospective study, based on the elaborate analysis using the new delineation guidelines of nodal level involvement of Radiation Oncology Therapy Group (RTOG) [16]. The aim of this study is to investigate the high-risk factors for metastasis of level IB and evaluate the feasibility of omission of elective irradiation to level IB in the subgroups without any of the high-risk factors. The objective of this study is to define the criteria of level IB-sparing radiation, in order to protect the function of submandibular glands and minimize late toxicities.

## Materials and methods

### Inclusion or exclusion criteria

Institutional Review Board approval was obtained for this retrospective review of patients. Consecutive patients with newly diagnosed, non-metastatic NPC, with complete imaging and treatment planning data, treated with definite IMRT in our center, were deemed eligible. Exclusion criteria were as follow: (1) surgery to primary tumor or cervical nodes prior to radiation (except for nasopharyngeal biopsy or fine needle aspiration of nodal disease); (2) who failed to complete the whole course of radiation; (3) who did not perform pretreatment MRI of head and neck in our center or the imaging data of pretreatment MRI were not traceable; (4) the treatment plan data were unavailable.

### Imaging analysis

One experienced radiologist and one experienced radiation oncologist in head and neck cancer reviewed all the imaging data. Any disagreement was solved by discussion. Cervical lymph nodes were defined as metastatic if the shortest axial diameter of jugulodigastric lymph node was ≥ 11 mm, or that of other lymph nodes was ≥ 10 mm, or there was a group of three or more lymph nodes in critical size [17]. The lateral retropharyngeal nodes were considered metastatic with their shortest diameter ≥ 5 mm. Any visible node in the median retropharyngeal group was considered malignant [18, 19]. In addition, the presence of extracapsular spread or central necrosis was also a sign of metastasis [17, 18, 20]. The involvement of nodal levels was evaluated according to the 2013 updated guidelines of RTOG [16]. All patients were restaged based on the 2010 AJCC staging system.

### Radiotherapy techniques

Patients were immobilized in the supine position with a thermoplastic mask. CT was performed after immobilization, obtaining 3-mm slices from the anterior clinoid process to the hyoid bone, and 5-mm slices from the hyoid bone to 2 cm below the sternoclavicular joint. The target volumes were outlined on each layer of the CT images on an IMRT workstation. The gross tumor volume (GTV) included primary tumor and metastatic lymph nodes. The details of the delineation of high-risk clinical target volume of primary tumor were described in our previous publication [4]. The high-risk CTV of cervical nodes should include bilateral coverage of levels II, III, VA and retropharyngeal nodes in N0 patients. For patients with metastatic cervical nodes of the upper neck (above cricoid cartilage), the low risk CTV should cover level IV and VB. For individuals with metastatic cervical nodes involving the lower neck, all the neck levels from II to V were defined as high-risk CTV. Elective level IB irradiation was decided by the attending physician, mainly based on these factors: maximal diameter of level IIA, extracapsular spread of level IIA, necrosis of level IIA, N stage, multiple levels involvement, maximal diameter of cervical nodes, as well as extracapsular spread or necrosis of cervical nodes. These factors were reasonably selected. The first three factors indicated the extent of involvement of level IIA, while the remaining reflected the burden of nodal metastasis and the risk of nodal relapse.

A margin of 3–5 mm around GTV and CTV should be added to account for the patient motion and set-up error. A simultaneous integrated boost method was used. 66 to 70.4 Gy was prescribed to primary tumor in 30–32 fractions. 66 Gy was delivered to the metastatic nodes in 30–32 fractions. The high-risk and low-risk CTV received 60 Gy and 54 Gy, respectively.

SMGs were contoured, but the SMG doses were not constrained during inverse treatment planning. Given the vicinity of CTV60, one half of SMG was within the isodose line 39Gy even if the ipsilateral level IB was not included in the CTV (Additional file 1: Figure S1). The mean doses of SMG were recorded retrospectively.

### Follow-up

Patients were followed up after completion of radiotherapy every 3 months in the first 2 years, then every 6 months from year 2 through year 5, and then annually. MRI of head and neck was required every 3 to 6 months in the first 3 years. Chest CT and abdominal sonography were performed at least every year. Bone scan or PET/CT was done when clinical indicated. Delayed xerostomia was evaluated in every follow-up, according to the patient’s subjective feeling and scored based on Common Terminology Criteria for Adverse Events 3.0 version grading system (CTCAE 3.0). Given that radiation-induced xerostomia faded with time and significantly recovered at 1 year post-IMRT [6], we compared the delayed xerostomia at 1 year after radiotherapy between the two arms – electively irradiation to level IB or not.

### Statistical analysis

Kaplan-Meier overall survival (OS), distant metastasis-free survival (DMFS) and local recurrence-free survival (LRFS) and regional recurrence-free survival (RRFS) were calculated. Event times for death/recurrence were calculated from the first day of radiation. Viable tumor cells with the same histological subtype at the time of salvage neck dissection or fine-needle aspiration were defined as regional failure. Baseline comparison was performed using independent t test and Chi-square test (or Fisher’s exact test, if indicated). The correlation of level IB metastasis and other factors were studied using Chi-square test (or Fisher’s exact test, if indicated). Logistic regression model (backward conditional) was used to investigate the independent predictive factor for level IB metastasis. In order to clarify the goodness of fit of Logistic model, we performed Hosmer-Lemeshow test. The *p* value of Hosmer-Lemeshow test > 0.05 indicated good estimation of Logistic model. Actual survival rates were compared by Log-rank test. Cox proportional-hazards models (enter method) were used to assess the effect of age, sex, T stage, N stage, nodal necrosis, chemotherapy, elective level IB radiation and boost irradiation to lymph nodes on regional control and overall survival. Since nodal extracapsular spread had certain correlation with elective level IB radiation, nodal extracapsular spread was used as classified factor in Cox model. Statistical significance was defined as *p* < 0.05 based on two-sided tests. All analyses were performed by SPSS software, version 22.0.

## Results

From January 2009 through December 2010, 869 consecutive patients with newly diagnosed, non-metastatic, biopsy-proven NPC completed the whole course of IMRT in our center. Among them, 46 patients received nodal excision prior to radiation, 185 patients underwent pretreatment MRI of nasopharynx or neck in other hospital and 106 patients were excluded due to data loss of pretreatment MRI or treatment plan backups. Therefore, the final study cohort consisted of 532 patients. The clinical characteristics were shown in Table 1. With a median follow-up of 51.5 (1.0–74.7) months, 47 patients experienced local recurrence, 22 had regional failures and 61 developed distant metastasis. At the time of analysis, 68 patients were dead. The 5-year estimated LRFS, RRFS and DMFS were 88.4%, 94.4% and 86.9%, respectively. The 5-year OS was 84.8%.

**Table 1:** Patient characteristics (*n* = 532)

		Number	Percentage (%)
Gender	Male	390	73
	Female	142	27
Age	Median	50	
	Range	11–80	
Pathology	WHO II-III	532	100
Stage	I	39	7
	II	179	34
	III	179	34
	IVA	55	10
	IVB	80	15
T	T1	176	33
	T2	173	33
	T3	124	23
	T4	59	11
N	N0	82	15
	N1	241	45
	N2	129	24
	N3	80	15
Elective IB Irradiation	Yes	232	44
	No	300	56
Radiation Boost	Primary tumor	32	6
	Nodal disease	43	8
Chemotherapy	Yes	451	85
	No	81	15

### Risk factors of level IB metastasis

Of 532 patients, 13 (2.4%) presented with level IB metastasis. The correlation of level IB metastasis and other factors were investigated by Chi-square test (or Fisher’s exact test, if indicated). More advanced N stage, bilateral nodal metastasis, level II involvement, level IIA involvement, level IIA metastasis with multiple levels involvement, the maximal axial diameter (MAD) of level IIA nodes >20 mm, MAD of neck lymph nodes > 30 mm, necrosis of level IIA nodes, extracapsular spread of level IIA correlated with level IB metastasis (*p* < 0.05, Table 2). There was no significant association between level IB metastasis and nasal or oropharyngeal involvement. Similarly, of nodal positive patients (*n* = 450), more advanced N stage, bilateral nodal metastasis, level IIA involvement, MAD of level IIA nodes > 20 mm, MAD of neck lymph nodes >30 mm and extracapsular spread of level IIA were associated with level IB metastasis (Table 2).

**Table 2:** Univariate analysis of risk factors of level IB metastasis in whole cohort and node-positive patients

	Level IB metastasis at diagnosis						
	All patients				Node + patients		
	Variable	(−), *n* = 519	(+), *n* = 13	*p* value	(−), *n* = 437	(+), *n* = 13	*p* value
N stage	N0	82	0	0.011*	0	0	0.029*
	N1	238	3		238	3	
	N2	121	8		121	8	
	N3	78	2		78	2	
Bilateral nodal metastasis	No	350	3	0.002*	268	3	0.005*
	Yes	169	10		169	10	
Nasal cavity involvement	No	402	10	1.000	342	10	1.000
	Yes	117	3		95	3	
Oropharyngeal involvement	No	491	13	1.000	409	13	1.000
	Yes	28	0		28	0	
Level II involvement	No	129	0	0.045*	47	0	0.378
	Yes	390	13		390	13	
Level IIA involvement	No	298	0	0.000*	216	0	0.000*
	Yes	221	13		221	13	
Level IIB involvement	No	156	2	0.362	74	2	1.000
	Yes	363	11		363	11	
Level III involvement	No	257	4	0.182	175	4	0.501
	Yes	262	9		262	9	
Level VA involvement	No	432	12	0.705	350	12	0.478
	Yes	87	1		87	1	
Level IVA involvement	No	461	11	0.649	379	11	0.687
	Yes	58	2		58	2	
Level IVB involvement	No	508	13	1.000	426	13	1.000
	Yes	11	0		11	0	
Level VB involvement	No	509	13	1.000	427	13	1.000
	Yes	10	0		10	0	
Level VC involvement	No	514	13	1.000	432	13	1.000
	Yes	5	0		5	0	
Retropharyngeal nodal (Level VIIA)metastasis	No	252	4	0.205	108	2	0.738
	Yes	267	9		326	10	
Level VIIB involvement	No	518	13	1.000	436	13	1.000
	Yes	1	0		1	0	
Level VIII involvement	No	507	13	1.000	425	13	1.000
	Yes	12	0		12	0	
Multiple levels involvement (II, III, VA, IVA, VIII)	<2	251	4	0.210	169	4	0.774
	≥2	268	9		268	9	
Level IIA metastasis with multiple levels involvement (II, III, VA, IVA, VIII)	No	355	4	0.007*	273	4	0.038*
	Yes	164	9		164	9	
MAD of IIA (mm)	≤20	388	4	0.001*	306	4	0.005*
	>20	131	9		131	9	
MAD of IIA (mm)	≤30	479	11	0.274	397	11	0.346
	>30	40	2		40	2	
MAD of neck nodes(mm)	≤30	288	3	0.020*	206	3	0.086
	>30	231	10		231	10	
Necrosis of level IIA nodes	No	463	9	0.048*	381	9	0.081
	Yes	56	4		56	4	
ES of level IIA nodes	No	398	4	0.001*	316	4	0.003*
	Yes	121	9		121	9	
MAD of IIA >20 mm or IIA necrosis	No	373	4	0.003*	291	4	0.014*
	Yes	146	9		146	9	
MAD of IIA >20 mm or IIA ES	No	363	3	0.001*	281	3	0.006*
	Yes	156	10		156	10	

Abbreviation: *MAD* maximal axial diameter, *ES* extracapsular spread

*p < 0.05

In multivariate analysis, bilateral nodal involvement (*p* = 0.045, HR = 4.056, 95% CI: 1.030–15.977), MAD of level IIA nodes >20 mm or extracapsular spread of level IIA nodes (*p* = 0.024, HR = 4.828, 95% CI: 1.227–18.996), were proven to be independent predictive factors of level IB metastasis at diagnosis (Table 3). Of nodal positive patients, MAD of level IIA nodes > 20 mm or extracapsular spread of level IIA nodes was demonstrated to be independent predictor of level IB metastasis by multivariate analysis (*p* = 0.034, HR = 4.287, 95% CI: 1.118–16.442, Table 3). In order to clarify the goodness of fit of logistic model, we performed Hosmer-Lemeshow test. For all patients, the Pearson Chi-squared value was 0.317 and the *p*-value was 0.854, which indicated the logistic model gave reliable estimation of observed incidence of level IB metastasis. Of node-positive patients, the Pearson Chi-squared value was 0.106 and the *p*-value was 0.948, which indicated excellent prediction of logistic model.

**Table 3:** Multivariate analysis of risk factors of level IB metastasis by Logistic regression model

Variable	Significance	HR	95% CI
All patients (*n* = 532)			
Bilateral nodal involvement	0.045*	4.056	1.030–15.977
MAD of IIA >20 mm or IIA ES	0.024*	4.828	1.227–18.996
Nodal positive patients (*n* = 450)			
Bilateral nodal involvement	0.062	3.601	0.938–13.826
MAD of IIA >20 mm or IIA ES	0.034*	4.287	1.118–16.442

Five variables with a *p*-value <0.05 in the univariate analysis of the whole cohort and node-positive cohort were included into Logistic regression model. The five variables are N stage, bilateral nodal involvement, MAD of IIA > 20 mm or IIA ES, MAD of IIA > 20 mm or necrosis of IIA, Level IIA metastasis with multiple levels involvement

*p < 0.05. Abbreviation: *MAD* maximal axial diameter; *ES* extracapsular spread

### Low-risk group

Based on previous analysis, the whole cohort of patients was divided into two subgroups. Those fulfilled either of risk factors (bilateral nodal involvement; MAD of level IIA nodes > 20 mm or extracapsular spread of level IIA nodes) were denoted high-risk group, while the rest were denoted low-risk group. Additional file: Table S1 compared the baseline characteristics by low-risk and high-risk groups.

**Fig. 1:**
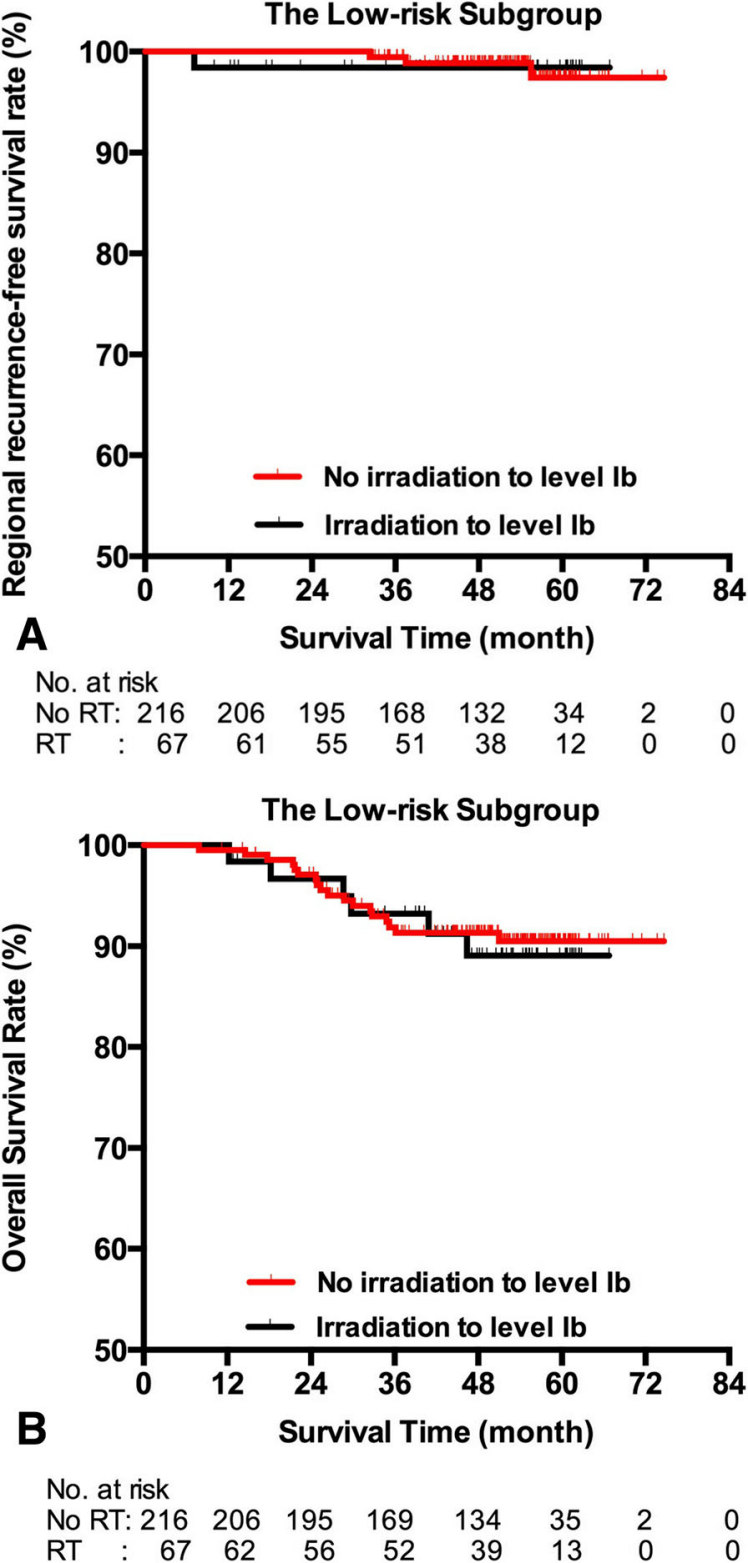
Regional recurrence-free survival (**a**) and overall survival (**b**) compared by elective irradiation to level IB in low-risk patients with nasopharyngeal carcinoma. There wasn’t significant difference of regional control (98.4% vs. 97.4%, *p* = 0.921) or overall survival (89.1% vs.90.5%, *p* = 0.798) between those with or without elective irradiation to level IB. The estimated 5-year RRFS for those with/without elective irradiation was 65.9 months (95% CI: 64.0–67.7 months), 73.9 months (95% CI: 73.1–74.6 months), respectively. The estimated 5-year OS for those with/without elective irradiation was 62.8 months (95% CI: 59.7–65.9 months), 70.3 months (95% CI: 68.3–72.2 months), respectively

Of the low-risk group (283), 216 did not received elective irradiation of level IB. Of note, patients with elective irradiation had more advanced N stage, higher proportion of nodal necrosis and extracapsular spread, higher proportion of chemotherapy, compared with those without elective irradiation (Additional file 1: Table S1). During the follow-up, four patients had neck relapse. Three of them did not receive level IB irradiation and only one recurrence located at level IB. The distributions of regional recurrence were shown in Additional file 1: Table S2. The percentage of level IB recurrence of those without elective irradiation was only 0.46%. There was no significant difference of regional control (98.4% vs. 97.4%, *p* = 0.921, Fig 1a), local control (90.4% vs. 90.4%, *p* = 0.599), DMFS (96.5% vs. 91.5%, *p* = 0.527) and OS (89.1% vs.90.5%, *p* = 0.798, Fig 1b) between those with or without elective irradiation of level IB. Elective level IB irradiation was not significant upon multivariate analysis both for regional control and overall survival, after adjusting the effect of age, gender, T stage, N stage, nodal necrosis, chemotherapy and boost irradiation to nodes (Table 4). N stage was an independent prognostic factor for regional control.

**Table 4:** Multivariate analyses of factors on regional recurrence-free survival and overall survival (the whole cohort of patients)

Factor	Regional control				Overall survival			
	*P* value	HR	95% CI		*P* value	HR	95% CI	
Low-risk group								
Age (≤ 50y; > 50y)	0.500	0.471	0.053	4.208	0.068	2.394	0.938	6.109
Gender (M/F)	0.760	0.657	0.045	9.654	0.920	0.956	0.392	2.331
T (T1/T2/T3/T4)	0.232	2.262	0.593	8.624	0.131	1.396	0.905	2.153
N (N0/N1/N2/N3)	0.008*	12.076	1.927	75.675	0.925	0.966	0.472	1.980
Nodal necrosis (no/yes)	0.722	0.530	0.016	17.542	0.147	2.155	0.763	6.088
Chemotherapy (no/yes)	0.182	0.101	0.003	2.924	0.528	0.699	0.229	2.128
Elective IB irradiation (no/yes)	0.941	1.122	0.054	23.274	0.799	1.143	0.408	3.206
Boost irradiation of lymph nodes (no/yes)	0.993	0.000	0.000	not reach	0.888	1.162	0.145	9.340
High-risk group								
Age (≤ 50y; > 50y)	0.198	1.909	0.714	5.104	0.058	1.798	0.980	3.299
Gender (M/F)	0.682	0.768	0.217	2.715	0.506	0.785	0.385	1.602
T (T1/T2/T3/T4)	0.648	0.879	0.504	1.531	0.285	1.190	0.865	1.637
N (N0/N1/N2/N3)	0.558	1.229	0.617	2.449	0.004*	2.080	1.269	3.411
Nodal necrosis (no/yes)	0.971	1.019	0.365	2.843	0.725	1.130	0.573	2.226
Chemotherapy (no/yes)	0.003*	0.029	0.003	0.299	0.000*	0.034	0.009	0.132
Elective IB irradiation (no/yes)	0.045*	8.540	1.050	69.473	0.243	0.690	0.371	1.285
Boost irradiation of lymph nodes (no/yes)	0.011*	3.793	1.351	10.645	0.906	1.058	0.413	2.710

*Indicated *p* < 0.05

In the subgroup of node-positive patients, no significant difference of 5-year regional control (98.3% vs. 96.0%, *p* = 0.798), local control (89.9% vs. 90.0%, *p* = 0.636), DMFS (96.3% vs. 92.8%, *p* = 0.677) and OS (88.4% vs. 91.5%, *p* = 0.547) was observed between those with or without elective level IB irradiation. Elective level IB irradiation was not significant upon multivariate analysis both for regional control and overall survival (Table 5). N stage was an independent prognostic factor for regional control and T stage was the independent prognostic factor for overall survival.

**Table 5:** Multivariate analyses of factors on regional recurrence-free survival and overall survival (node-positive patients of low-risk group)

Factor	Regional control				Overall survival			
	*P* value	HR	95% CI		*P* value	HR	95% CI	
Low-risk group								
Age (≤ 50y; > 50y)	0.486	0.461	0.052	4.077	0.707	1.225	0.426	3.521
Gender (M/F)	0.735	0.630	0.044	9.121	0.920	1.057	0.361	3.092
T (T1/T2/T3/T4)	0.298	2.064	0.527	8.089	0.046*	1.776	1.010	3.125
N (N1/N2/N3)	0.015*	10.370	1.577	68.215	0.302	1.578	0.663	3.755
Nodal necrosis (no/yes)	0.718	0.535	0.018	15.953	0.143	2.267	0.758	6.782
Chemotherapy (no/yes)	0.177	0.105	0.004	2.760	0.295	0.449	0.100	2.009
Elective IB irradiation (no/yes)	0.971	1.056	0.055	20.353	0.452	1.547	0.496	4.832
Boost irradiation of Lymph nodes (no/yes)	0.993	0.000	0.000	not reach	0.773	1.374	0.158	11.972

*Indicated *p* < 0.05

### High-risk group

Of the high-risk group (249), 84 did not received elective irradiation of level IB. It was noteworthy that patients with elective irradiation of level IB had a higher proportion of nodal necrosis and extracapsular spread. During the follow-up, there were 18 nodal recurrences. However, the majority (17/18) of them belonged to those with elective irradiation and no relapse located at level IB (Additional file 1: Table S2). Those with elective irradiation of level IB tended to have a poorer regional control (87.3% vs. 98.1%, *p* = 0.018). There was no significant difference of local control (86.0% vs. 84.5%, *p* = 0.108), DMFS (81.8% vs. 78.0%, *p* = 0.465) and OS (81.2% vs.72.4%, *p* = 0.156) between those with or without elective irradiation of level IB. Elective level IB irradiation was marginal significant for regional control, but not for overall survival, upon multivariate analysis incorporating age, gender, T stage, N stage, nodal necrosis, chemotherapy and boost irradiation to nodes. N stage and chemotherapy were demonstrated to be independent prognostic factor for overall survival (Table 4).

### Dose of SMG and delayed xerostomia

Elective irradiation of level IB increased the mean dose of SMG, regardless of left (6317+/−526 vs. 4576+/− 2232 cGy, *p* = 0.000) or right side (6368+/−210 vs. 4610+/− 2327 cGy, *p* = 0.000). The mean dose of SMG exceeded 50Gy in above 75% (78.8%, left SMG; 79.1%, right SMG) of cases without ipsilateral level IB irradiation. In terms of delayed xerostomia, there was no significant correlation between elective level IB irradiation and the grade of patient-reported xerostomia at 1 year post-IMRT (*p* = 0.296, Additional file 1: Table S3).

## Discussion

Lymph node metastasis in NPC follows an orderly pattern along deep cervical lymph nodes. The most commonly involved regions include level II (70–87.4%) and retropharyngeal lymph nodes (69–75.1%), followed by level III, level IV and level V [21, 22]. The probability of “skip” metastasis is quite low (varies between 0.5–7.9%) [21]. The incidence of level IB metastasis in NPC is very low, varies from 2.2% to 4.3% [18, 20–22] (the rate of our study was 2.4%). Level IB nodes receive efferent lymphatic from the level IA, the lower nasal cavity, the hard and soft palate, the maxillary and mandibular alveolar ridges, the cheek, the upper and lower lips, and most of the anterior tongue [16]. Level IB nodes do not receive direct drainage from nasopharynx. Hence, it is reasonable not to include level IB in the CTV of all patients with NPC.

In recent study including 3100 cases of NPC [22], all patients with level IB node involvement were simultaneously accompanied with level II lymphadenopathy. It is reasonable to assume that the enlargement of level II nodes blocks the routine pathway and causes reflux to level IB. Several studies [11, 23] further proved this hypothesis. Yuan et al. [23] reported that the diameter of level II was the only significant factor associated with level IB lymphadenopathy in multivariate analysis in NPC. Zhang et al. [11] demonstrated that the diameter of level IIA > 20 mm or extracapsular spread of level IIA was an independent predictive factor for level IB metastasis. In consistence with previous study, our research indicated that the diameter of level IIA > 20 mm or extracapsular spread of level IIA was significantly associated with level IB lymphadenopathy. The extracapsular spread causes involvement of carotid sheath, which was proven to correlate with level IB metastasis in another study by Yi et al. [24]. Taken together, the remarkable enlargement and spread of level IIA may result in blockage of routine drainage pathway, which causes reflux to its afferent lymph node-level IB. Bilateral involvement of cervical nodes were proven to be independent factor both in the present study and the report by Zhang et al. [11] as well. Given the possibilities of bilateral lymphatic drainage when nasopharynx tumor crossed the midline, bilaterally lymphadenopathy further added to burden of level II, therefore, increased the possibility of reflux to level IB.

The correlation between oropharyngeal involvement and level IB lymphadenopathy was inconsistently reported. Oropharyngeal involvement was not an independent predictive factor in the study of Yi [24] and Yuan [24] et al., but was significant in the report by Zhang [11] et al. Wang [22] et al. reported that about one third of cases with level IB involvement accompanied with oropharyngeal infiltration. In the present study, oropharynx invasion was not significantly associated with level IB metastasis. It is probably due to the limited number of cases with oropharyngeal involvement and the underpowered test. The correlation between oropharyngeal involvement and level IB lymphadenopathy warrants further investigation. In addition, the nasal cavity involvement was not an independent predictive factor for level IB metastasis in the present study, which was similarly observed in previous studies [11, 23, 24]. It is generally accepted that level IB receives lymphatic drainage of the anterior one third of nasal cavity [24, 25]. However, tumor generally involves posterior one third of nasal cavity in NPC.

Furthermore, our study incorporated elaborate analysis of various neck nodal involvements into the risk-analysis of level IB metastasis, using the updated delineation guidelines of neck nodal levels [16], which was not reported in previous analyses [11, 23]. Our study revealed that level IIA accompanying with multiple adjacent levels involvement was associated with level IB metastasis in univariate analysis, but did not reach statistically significant level in multivariate analysis. Similarly, in another investigation [23], four or more lymphatic drainage regions involved reached a marginal level (*p* = 0.05) in univariate analysis, but was not at statistically significant level in multivariate analysis. In addition, Yi et al. [24] demonstrated that level II/III/IV involvement was independent risk factor of level IIA metastasis in a cohort of patients treated with three-dimensional conformal radiotherapy. Given that multiple adjacent nodal levels involvement may block the level IIA efferent lymphatic drainage, it is reasonable to postulate that this may cause increased risk of reflux to level IB. Further investigation with a larger sample size is requested to confirm this postulation. Hence, we recommend taking this factor into consideration when evaluating the metastatic risk of level IB.

Given the low incidence of metastasis of level IB in NPC, it is reasonable not to include level IB in the CTV in the low-risk subgroup of level IB metastasis. A few studies have evaluated the feasibility of omitting irradiation to level IB in NPC. Chen et al. [26] retrospectively investigated a cohort of 120 patients treated with level IB-spared IMRT. During 54 months of follow-up, four patients had regional recurrence and none located in level IB. However, the details of cervical nodal involvement were not mentioned in this study. Zhang et al. [11] investigated a cohort of 1438 patients treated with IMRT and demonstrated the high-risk factors for level IB lymphadenopathy included the diameter of level IIA nodes ≥20 mm and/or level IIA nodes with extracapsular spread, positive bilateral nodes or oropharynx involvement at diagnosis. Patients without these risk factors were defined low-risk and none of them (0/904) experienced regional recurrence at level IB. Level IB irradiation was not an independent prognostic factor for locoregional control, distant metastasis and overall survival in low-risk subgroup. Similarly, Yi et al. [24] developed a risk scoring models to predict level IB metastasis and divided patients into low-risk and high-risk subgroups. The model included factors such as the carotid sheath involvement, maximal diameter of the neck lymph node (≥ 20 mm) and involvement of level II/III/IV lymph nodes. In the low-risk group, level IB irradiation had no significant influence on overall survival, locoregional control and distant metastasis. Of note, patients in the prognostic study received three-dimensional conformal radiotherapy [24].

In consistence with previous studies, our research defined the low-risk group as those without any of the following criteria: the diameter of level IIA nodes > 20 mm and/or level IIA nodes with extracapsular spread, positive bilateral cervical lymph nodes. In the low-risk subgroup without level IB irradiation, only one patient experienced regional recurrence at level IB. The percentage of level IB recurrence of those treated with level IB-sparing IMRT was only 0.46% (1/216). Level IB irradiation has no significant impact on regional control, distant metastasis and overall survival in the low-risk patients.

Interestingly, of the high-risk subgroup, those with elective irradiation of level IB tended to have a poorer regional control. Further analysis revealed that those with elective irradiation of level IB had a higher percentage of nodal necrosis and extracapsular spread, as well as greater maximal diameter of cervical nodes (data not shown, 43.06+/− 16.76 vs. 38.58 +/− 17.07 mm, *p* = 0.049). Nodal necrosis [27] and maximal diameter [28, 29] were proven to be prognostic factor for regional relapse, thus partially explaining the intriguing results. In addition, level IB was electively included in the CTV by the attending physician’s decision, based on clinical and imaging characteristics. Therefore, patients with more adverse prognostic factors, tended to be treated with level IB irradiation.

In the present study, elective omission of level IB irradiation significantly reduced the mean dose of ipsilateral SMG, however, did not transfer to improvement of patient-reported xerostomia. One explanation is that there was no mandatory restriction of SMG dose during inverse planning in our institution at that time. According to a dose-effect study, the incidence of grade IV xerostomia dramatically increases once the mean dose of SMG exceeds a threshold of 39Gy. The saliva secretion of SMG could recover over time if the mean dose of SMG is below 39Gy [12]. Once the mean dose of SMG exceeds 50Gy, the probability of grade IV toxicity is above 80% [12]. In our study, more than 78% of patients received more than 50Gy to SMG even if the ipsilateral level IB was not included in the radiation volume. Thus, despite elective omission radiation of level IB, the improvement of patient-reported xerostomia was mild. Another reason is that several factors have influence on xerostomia, such as bilateral involvement of level II, oropharyngeal infiltration of bulky tumor, mean dose of parotid, mean dose of oral cavity where minor salivary glands disperse. Gensheimer et al. [9] has shown that with careful dose constraint of parotid (mean dose < 24 Gy) and contralateral SMG (mean dose < 39 Gy), the contralateral SMG-sparing technique caused a notable and durable improvement of xerostomia. A future validation study that restrains the dose of parotid and spares SMG is warranted to confirm the benefit of elective omission of level IB in NPC.

A limitation of our study was the retrospective design. The incidence of level IB metastasis at diagnosis was relatively low. However, multivariate analysis indicated the significant risk factors for level IB involvement, which were well consistent with previous studies. Another limitation was no routine dose constraint for spared SMG in our institution at that time, which hampered the translation into the improvement of patient-subjective xerostomia.

## Conclusion

In conclusion, our study demonstrated the risk factors for level IB metastasis were the diameter of level IIA nodes > 20 mm and/or level IIA nodes with extracapsular spread, positive bilateral cervical lymph nodes. For patients without either of the risk factors, omission of elective irradiation to level IB did not result into significant difference of regional control, DMFS and OS, but notably reduced the mean dose of ipsilateral SMG. It may be feasible to omit the radiation of level IB in the patients with low risk of level IB metastasis. A prospective study with careful dose constraint of SMGs and parotid is warranted to confirm the benefit.

## Additional file

Additional file 1:
**Table S1.** Comparison of clinical factors between high-risk and low-risk subgroups. **Table S2.** Distribution of regional recurrence by risk-stratified subgroups. **Table S3.** The grades of xerostomia by elective irradiation to level IB. **Figure S1.** Isodose distribution of submandibular glands. (PDF 240 kb)

## Abbreviations

CTV: Clinical tumor volume
DMFS: Distant metastasis-free survival
GTV: Gross tumor volume
IMRT: Intensity-modulated radiation therapy
LRFS: Local recurrence-free survival
MAD: Maximal axial diameter
NPC: Nasopharyngeal carcinoma
OS: Overall survival
RRFS: Regional recurrence-free survival
SMG: Submandibular gland
