# Supplementary material for: The feasibility analysis of omission of elective irradiation to level IB lymph nodes in low-risk nasopharyngeal carcinoma based on the 2013 updated consensus guideline for neck nodal levels
Source: Radiat Oncol. 2017 Aug 18;12:137. doi: 10.1186/s13014-017-0869-x (PMC5561583; doi:10.1186/s13014-017-0869-x)
Supplement: Additional file 1: — Table S1. Comparison of clinical factors between high-risk and low-risk subgroups. Table S2. Distribution of regional recurrence by risk-stratified subgroups. Table S3. The grades of xerostomia by elective irradiation to level IB. Figure S1. Isodose distribution of submandibular glands. (PDF 240 kb) [file 13014_2017_869_MOESM1_ESM.pdf]

Additional file

Table S1. Comparison of clinical factors between high-risk and low-risk subgroups.
Table S2. Distribution of regional recurrence by risk-stratified subgroups.
Table S3. The grades of xerostomia by elective irradiation to level IB.
Figure S1. Isodose distribution of submandibular glands.

Table S1. Comparison of clinical factors between high-risk and low-risk subgroups

Variable	Low-risk			High-risk		
	IB RT (-)	IB RT (+)	p value	IB RT (-)	IB RT (+)	p value
Age	51.7+/-11.4	50.5+/-111.7	0.281	48.7+/-12.0	46.2+/-12.4	0.134
Gender M/F	157/59	45/22	0.382	63/21	125/40	0.895
T stage T1/T2/T3/T4	79/69/36/32	23/22/18/4	0.111	23/25/23/13	51.57/47/10	0.112
N stage N0/N1/N2/N3	79/128/0/9	3/57/0/7	0.000*	0/16/49/19	0/40/80/45	0.336
Nodal necrosis -/+	186/30	45/22	0.000*	45/39	65/100	0.033*
Nodal ES -/+	159/57	26/41	0.000*	19/65	21/144	0.044*
Chemotherapy -/+	73/143	4/63	0.000*	2/82	2/163	0.605
Boost RT to LN -/+	206/10	65/2	0.738	76/8	142/23	0.318

* p< 0.05.

Abbreviation: ES= extracapsular spread. RT=radiation. LN= lymph nodes.

Table S2. Distribution of regional recurrence by risk-stratified subgroups

Patients and groups	Distribution of regional recurrence
Low-risk group	
No elective level Ib irradiation	
Patient #1	N.A.
Patient #2	Left IB
Patient #3	Left IIB
Elective level Ib irradiation	
Patient #4	Right VB
High-risk group	
No elective level Ib irradiation	
Patient #5	Right IIB
Elective level Ib irradiation	
Patient #6	Right IIB, III
Patient #7	Right IIB, III
Patient #8	Right IIA
Patient #9	Left retropharyngeal, IIA, IIB, III
Patient #10	Left retropharyngeal, IIB
Patient #11	N.A.
Patient #12	Right III, IVA, IVB, VIII
Patient #13	Left IIA
Patient #14	Right IIB, III
Patient #15	Right IIB, IVA
Patient #16	Right IIA
Patient #17	Left IVA
Patient #18	Bilateral IIA, IIB, III; Right IVA
Patient #19	Left IIB, III
Patient #20	Left VIII
Patient #21	Left III
Patient #22	Right retropharyngeal, IIA, IIB, III, VII, VIII

Table S3. The grades of xerostomia by elective irradiation to level IB at one year after IMRT

	Elective irradiation of level IB		
Grade of xerostomia	No	Unilateral	Bilateral
0	166	72	54
1	6	0	4
2	118	60	33
3	10	5	4
Total	300	137	95

Figure S1

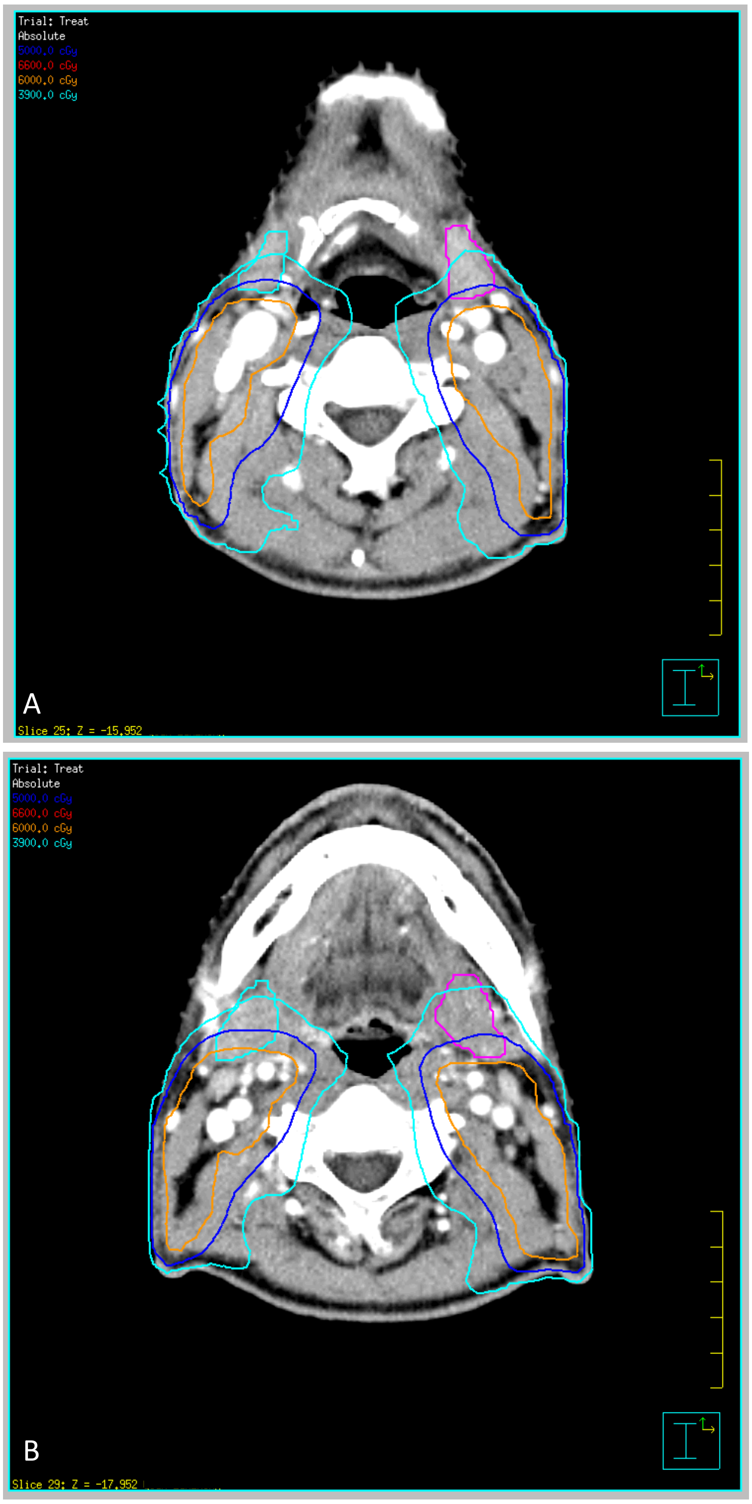

Figure S1 Isodose distribution of submandibular glands (A and B)

It is noted that the submandibular glands were contoured and half of the glands were covered by 39Gy isodose line. Two thirds of the glands were covered by 50Gy isodose line. Isodose lines included 39Gy isodose line (light blue), 50Gy isodose line (dark blue), 60Gy isodose line (orange) and 66Gy isodose line (red).
